# Supplementary material for: X-ray Irradiation Reduces Live Aspergillus flavus Viability but Not Aflatoxin B1 in Naturally Contaminated Maize
Source: Toxins (Basel). 2024 Jul 25;16(8):329. doi: 10.3390/toxins16080329 (PMC11359306; doi:10.3390/toxins16080329)
Supplement: Supplementary file 1 [file toxins-16-00329-s001.zip › SI_Figures_X-ray_Irradiation_2024-07-19.pdf]

a.

b.

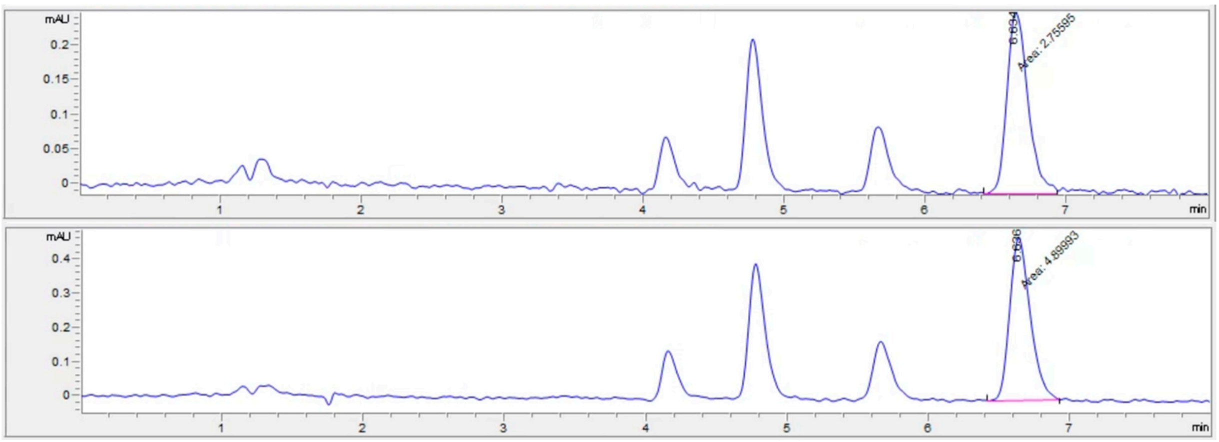

Supplementary Figure S1. HPLC AFB1 detection limits using certified reference standard diluted with mobile phase. (a). LOD of AFB1 standard mix at 5 ppb. (b) LOQ of AFB1 standard mix at 20 ppb. The peak order is: AFG2, AFG1, AFB2, and AFB1.
