# Supplementary material for: X-ray Irradiation Reduces Live Aspergillus flavus Viability but Not Aflatoxin B1 in Naturally Contaminated Maize
Source: Toxins (Basel). 2024 Jul 25;16(8):329. doi: 10.3390/toxins16080329 (PMC11359306; doi:10.3390/toxins16080329)
Supplement: Supplementary file 1 [file toxins-16-00329-s001.zip › SI_Tables_X-ray_Irradiation_2024_07-19.pdf]

Supplemental Table S1. Dilution plating counts for modified rose bengal media.

**X-ray dosage**	**Sample**	**CFU by Dilution for Modified Rose Bengal Media**					
		**10^1**	**10^2**	**10^3**	**10^4**	**10^5**	**10^6**
**0**	**1**	TMTC	102	6	0	0	0
	**2**	TMTC	127	2	0	0	0
**1**	**3**	9	0	0	0	0	0
	**4**	3	0	0	0	0	0
**1.5**	**5**	4	1	0	0	0	0
	**6**	1	0	0	0	0	0
**2**	**7**	1	0	0	0	0	0
	**8**	1	0	0	0	0	0
**2.5**	**9**	0	0	0	0	0	0
	**10**	0	0	0	0	0	0
**3**	**11**	0	0	0	0	0	0
	**12**	0	0	0	0	0	0
**Positive control**	**13**	104	4	0	0	0	no plate
**Negative control**	**14**	4	0	0	0	0	no plate

*TMTC = too many to count

Supplemental Table S2. Dilution plating counts for potato dextrose agar.

X-ray dosage	Sample	CFU by Dilution for Potato Dextrose Agar			
		10^1	10^2	10^3	10^4
0	1	TMTC	TMTC	15	2
	2	TMTC	59	12	1
1	3	11	0	0	1
	4	9	2	1	0
1.5	5	3	0	0	0
	6	11	0	0	0
2	7	0	0	0	0
	8	0	0	0	0
2.5	9	0	0	0	0
	10	1	0	0	0
3	11	0	0	0	0
	12	0	0	0	0
**Positive control**	13	TMTC	17	4	no plate
**Negative control**	14	44	6	1	no plate

*TMTC = too many to count

Supplemental Table S3. HPLC standard curve, LOD and LOQ calculation

Retention time	Peak Area	Expected concentration	Calculated concentration (ppb)	Recovery	Recovery within 20%?
6.66053248	1.10705614	5	10.93	2.19	LOD - No
6.64309168	1.23129785	5	11.28	2.26	No
6.64463759	1.21024346	5	11.22	2.24	No
6.63428021	2.75594592	10	15.59	1.56	No
6.63135338	2.79567719	10	15.7	1.57	No
6.63322687	2.69740534	10	15.42	1.54	No
6.63631821	4.89992571	20	21.65	1.08	LOQ - Yes
6.63766098	5.0501895	20	22.08	1.1	Yes
6.63619471	4.89412451	20	21.63	1.08	Yes
6.63146925	14.3001995	50	48.23	0.96	Yes
6.63039064	14.6635542	50	49.26	0.99	Yes
6.63088322	14.4401999	50	48.63	0.97	Yes
6.62661886	30.4900856	100	94.02	0.94	Yes
6.62944984	30.4867497	100	94.01	0.94	Yes
6.63227797	30.6167908	100	94.38	0.94	Yes
6.61981583	64.1263809	200	189.14	0.95	Yes
6.62076235	63.9958916	200	188.77	0.94	Yes
6.62212753	63.6446037	200	187.78	0.94	Yes
6.59236527	176.195496	500	506.06	1.01	Yes
6.59176254	175.056793	500	502.84	1.01	Yes
6.59115648	176.308502	500	506.38	1.01	Yes

$R^2 = 0.9989$
